# Broad, subjective, relative: the surprising folk concept of basic needs

**DOI:** 10.1007/s11098-023-02080-9

**Published:** 2023-12-21

**Authors:** Thomas Pölzler, Tobu Tomabechi, Ivar R. Hannikainen

**Affiliations:** 1 Department of Philosophy, University of Graz, Attemsgasse 25/II, 8010 Graz, Austria; 2 Department of Social Psychology, University of Tokyo, 7-3-1 Hongo, Bunkyo-Ku, Tokyo, 113-0033 Japan; 3 Department of Philosophy I, University of Granada, Campus de la Cartuja, 18011 Granada, Spain

**Keywords:** Basic needs, Experimental philosophy, Normative theory, Conceptual analysis

## Abstract

Some normative theorists appeal to the concept of basic needs. They argue that when it comes to issues such as global justice, intergenerational justice, human rights or sustainable development our first priority should be that everybody is able to meet these needs. But what are basic needs? We attempt to inform discussions about this question by gathering evidence of ordinary English speakers’ intuitions on the concept of basic needs. First, we defend our empirical approach to analyzing this concept and identify a number of its potential features. Then we present three preregistered empirical studies that were conducted to investigate the extent to which ordinary speakers endorse these features. The studies yield convergent evidence for the following three claims: (1) ordinary speakers sometimes apply the concept of basic needs to necessities for a flourishing (not just a minimally decent) life, (2) most ordinary speakers attribute at least some degree of subjectivity to the concept, and (3) most ordinary speakers attribute at least some degree of relativity to the concept. We discuss the implications of these findings for philosophical analyses of *basic needs*.

## Introduction

Normative theorists regularly appeal to the concept of basic needs. For example, they have argued that this concept is relevant to determining what we owe people who live in developing countries (e.g., Brock, 2009; Doyal & Gough, 1991) or in the future (e.g., Meyer & Pölzler, 2022; Wolf, 2009), to justifying human rights (e.g., Brock, 2005; Miller, 2007) and to defining the idea of sustainable development (e.g., WCED, 1987).

For any needs-based normative theory to succeed it must involve a clear, well-supported and operationalizable account of what basic needs are. In the 1980s and 1990s this issue sparked lively debate, as scholars developed a number of different definitions of basic needs (e.g., Braybrooke, 1987; Doyal & Gough, 1991; Frankfurt, 1988; Wiggins, 1998). However, with the advent of the capabilities approach (Sen, 1992, 1999), the concept somewhat fell out of favor again, and these discussions faded out before reaching full maturity. Today it therefore remains unclear and controversial how appeals to basic needs in normative theories are to be understood (as also pointed out, e.g., by Gasper, 2007; Hassoun, 2021).

Our goal in this paper is to revive the debate about the meaning of the concept of basic needs in normative contexts. So far this meaning has primarily been stipulated in relation to theoretical or practical aims or informed by theorists’ introspective observations about their own or ordinary speakers’ conceptual intuitions. The approach employed in this paper, in contrast, is empirical. We contribute to analyses of *basic needs* in normative contexts by providing empirical evidence on ordinary speakers’ intuitions about this concept—similarly to how experimental philosophers have recently gathered such evidence in exploring various other concepts, such as *knowledge*, *causation*, *intentional action* or *moral responsibility* (e.g., Hitchcock & Knobe, 2009; Knobe, 2003; Machery et al., 2017; Nichols & Knobe, 2007).^1^

First, we will briefly defend our empirical approach to analyzing *basic needs*. Then we will identify and explain a number of potential features of the concept. Three preregistered empirical studies were conducted to investigate the extent to which ordinary English speakers endorse these features.^2^ Study 1 tested participants’ reactions to hypothetical cases in which a potential basic need lacks each of the features. Study 2 asked participants to report whether these same features are either contingently or necessarily true of basic needs. Finally, Study 3 asked participants to freely define *basic needs* in their own words.

After having reported and discussed these studies, we will reflect on what these results suggest for philosophical analyses of the concept of basic needs. We will argue that they provide some initial evidence for broader, and against strongly objectivist and universalist, analyses.

## The relevance of folk intuitions

This paper is based on the claim that evidence about ordinary speakers’ intuitions can contribute to justifying philosophical analyses of the concept of basic needs. In what follows we will explain and provisionally support this claim.

In the sense in which the term is used here, intuitions are dispositions to apply a concept in certain ways. These dispositions are pre-theoretical, i.e., they must not have been derived from any theory that the speaker holds (Kauppinen, 2007; Loeb, 2008). Thus, a person can be attributed the intuition that *basic needs* exemplifies some feature *x* if and only if that person is disposed to apply the concept to things that have *x*, and to refrain from applying the concept to things that lack *x*, without having derived this disposition from theories about basic needs or related matters.

Many philosophers are internalists about the meaning of philosophical concepts (Jackson, 1998; Kauppinen, 2007; Loeb, 2008). Hence, they would readily accept that ordinary speakers’ intuitions in the above sense can contribute to justifying analyses of basic needs. This claim has sometimes been endorsed explicitly (e.g., Brock, 2005; Copp, 1998; Gasper, 2007). For example, Braybrooke (1987) writes:

What […] needs are, met or unmet, is to be determined by inquiring what the concept of needs means to the people who have the concept. […] my findings are arrived at as hypotheses about how speakers of English use the term “needs.” Their linguistic practice in using this term to debate social issues, with the nuances of their practice, serves as the focus of my thinking about the concept. (Braybrooke, 1987: 39)

In contrast, some theorists with a background in the social sciences^3^ have instead treated *basic needs* as a technical concept. These theorists have assumed that the concept’s meaning is, to a large extent, to be freely stipulated by theorists, based on theoretical and practical aims as well as on the normative context under consideration. The meaning need not match the folk concept to any particular extent (e.g., Hicks & Streeten, 1979; Stewart, 1985; Streeten & Burki, 1978)—an approach that is related to what philosophers nowadays call “conceptual engineering” (e.g., Cappelen, 2018).

Note that the above two approaches—the folk psychological approach and the stipulative approach—are not mutually exclusive. One can claim that folk intuitions should inform analyses of the concept of basic needs to a greater or lesser degree. In fact, we take it that with regard to many theoretical and practical aims and normative contexts some combination of both approaches will be preferable. In what follows, we will therefore not purport to argue that ordinary speakers’ intuitions should *fully* or even only *dominantly* determine analyses of the concept of basic needs. We will only provide the contours of five arguments for the claim that, depending on one’s aims and normative context, such intuitions will often be at least *somewhat* relevant.^4^

First, many needs-theorists have explicitly capitalized on the fact that the concept is part of ordinary language. Appeals to these needs have been said to be particularly promising because the concept is more widely accepted across cultures than some alternative concepts, such as *human rights* (Miller, 1999); because people understand it more easily than some alternative concepts (Wiggins, 1998), such as (*basic*) *capabilities*; because to say that someone has a basic need for something has a greater motivational or rhetorical force (Pinzani, 2013; Reader, 2007; Schuppert, 2013); and so on. If the concept were analyzed without any regard for how it is used by ordinary speakers then these advantages (which are generated by this usage) could presumably not be claimed. The appeal of needs-based normative theories would diminish.

Second, the theoretical aim of appeals to basic needs often involves specifying a threshold of well-being that each person should be able to reach (e.g., Meyer & Roser, 2009; Meyer & Pölzler, 2022; Miller, 1999). The concept of basic needs has been claimed to be particularly well-suited to such a specification as it seems to entail a non-arbitrary difference between a life that is minimally decent and one that is not. For example, while it seems arbitrary that having 30% of one’s preferences satisfied, earning $2 a day, or having 20 valued capabilities is enough (in terms of justice), it does not seem as arbitrary that being able to meet one’s needs for food, water, shelter, etc. is enough (e.g., Benbaji, 2005; Miller, 2007). Again, however, for needs-theorists to be able to claim this advantage it seems that their analyses must at least somewhat draw on the folk concept of basic needs. Free stipulations would render threshold-specifications in terms of basic needs as arbitrary as those based on alternative concepts (Meyer & Pölzler, 2022).

Third, on a plausible account of the purpose of normative theorizing this theorizing should guide people in dealing with practical problems that occur in the non-ideal world that they inhabit. For such guidance to be possible, normative researchers need to make sure that most people can be brought to accept their theories (e.g., Farelly, 2007; Miller, 2003)—which is more likely for theories that are based on an ordinary interpretation of *basic needs* (which they themselves share and immediately understand) than on a revisionary meaning that is stipulated and explained by experts (Brock, 2005). More specifically, expert-definitions may be perceived as a form of paternalism or elitism: “How can *they* claim to know what *we* mean when we say that we need something?”.

Fourth, analyses of basic needs that remain unconstrained by folk usage are more vulnerable to biases. Experts typically occupy a very peculiar social position, e.g., they rank much higher than the average person in terms of income, wealth, education, and are more likely to hold left-leaning political views (for philosophers see, e.g., Bourget & Chalmers, 2020; Schwitzgebel et al., 2021). It is possible that these social and demographic characteristics affect the outcome of their analyses in undue ways. For example, in a previous study (Pölzler & Hannikainen, 2022) we found that participants in higher income brackets endorsed narrower conceptions of *basic needs* than those in lower brackets—possibly because those in higher brackets are more likely to be providers and those in lower brackets are more likely to be recipients of means to needs satisfaction. Considering how other people use the concept can help to avoid such potential biases that could arise from experts’ peculiar social position (Miller, 2020).

Fifth, semantic externalism—the view that the meaning of concepts is determined by factors that are fully external to people’s minds—is unlikely to apply to *basic needs*. Arguments for this view are most plausible for concepts that refer to things that share some inner nature and can be pointed to without knowledge of this nature. For example, Putnam (1975) argued that the fact that the concept of water refers to H_2_O is ultimately determined by water’s actual nature (in particular, by its chemical structure), and hence it does not matter what ordinary speakers are pre-theoretically disposed to apply this concept to. In contrast, it is doubtful that all of the things that are needed in a basic sense share some inner nature beyond human categorization (think, e.g., of how different food and education are); and even if they did, many of these things could not easily be pointed to without knowledge of this nature (e.g., basic needs for air or health).

Again, the above arguments for considering folk intuitions do not purport to refute the stipulative approach. Revisions by experts will, to smaller or larger extents, be justified or even required in many contexts. For example, it seems perfectly plausible to us to claim that theorists should improve on the folk concept of basic needs by removing inconsistencies, making vague aspects more precise and reducing problematic ontological commitments (e.g., Andow, 2020; Cappelen, 2018); and that they may adjust the concept to fit their theoretical and practical aims and the particular normative context that they are addressing. The only thing that we have attempted to show in this section is that ordinary speakers’ conceptual intuitions should be at least *somewhat* accommodated by *some* analyses of *basic needs*.

## Potential features of basic needs

What, then, is the content of the folk concept of basic needs? While philosophers have traditionally addressed this question by drawing on their own observations and interpretations (e.g., Braybrooke, 1987; Brock, 2005; Copp, 1998), we assume that there can be value in certain kinds of scientific investigations of ordinary speech as well (e.g. Hannon, 2018; Pölzler, 2018).

Conceptual knowledge can be implicit in the sense that speakers need not be consciously aware of, or able to articulate, their intuitions (Hampton, 1999). Studies 1 and 2 of our research thus will not ask participants about the features that they spontaneously attribute to *basic needs* but rather request that they rate a series of candidate features. These features were obtained by conducting a literature search on normative theories that involve analyses of the concept of basic needs, using online platforms such as *PhilPapers* and *Google Scholar.* We found that while there was no unanimity with regard to any feature, 13 features have been widely discussed as potential constituents of the concept’s *analysans*. These features can be divided into (1) goal features and (2) non-goal features.

Normative researchers often regard (basic) needs as necessities for goal-achievement: to say that a person needs *x* means that *x* must be the case in order for some *y* to be the case (Fletcher, 2018). Goal-features pertain to the *y* in this formula. Six goals have often been claimed to be necessary for, or characteristic of, basic needs (with goals F2, F3 and F4 often being treated as specifications of goal F1). In particular, normative theorists have claimed that to say that *P* has a basic need for *x* entails that if *P* does not have/realize/ etc. *x* …

1. **Serious Harm:** … then P is seriously harmed (see, e.g., Frankfurt, 1988; Copp, 1998; Thomson, 2005; Wiggins, 1998)
2. **Autonomy:** … then P can no longer be sufficiently autonomous (which constitutes serious harm) (e.g. Copp, 1998; Doyal & Gough, 1991; Meyer & Pölzler, 2022).
3. **Rationality:** … then P does no longer have sufficient rational agency (which constitutes serious harm) (e.g., Brock, 2005; Schuppert, 2013).
4. **Social Functioning Harm:** … then P can no longer sufficiently function as a member of society (which constitutes serious harm) (e.g., Braybrooke, 1987; Miller, 1999).
5. **Survival:** … then P cannot survive (considered by Schuppert, 2013).
6. **Flourishing:** … then P cannot flourish (considered by Stewart, 1985).

Besides goals, normative researchers have also analyzed basic needs in reference to a variety of non-goal features. In particular, many of these researchers have claimed that to say that P has a basic need for x…

(F7) **Irreducibility:** … is not equivalent to saying that P has any kind of preference for x (e.g., Hamilton, 2003; Pinzani, 2013; Reader, 2007; Schuppert, 2013; Wiggins, 1998).
(F8) **Normativity:** … entails that states or persons have a *pro tanto* reason to enable P to have/be/realize/etc. x (e.g., Braybrooke, 1987; Reader and Brock, 2004; Doyal & Gough, 1991; Thomson, 1987; Wiggins, 1998).
(F9) **Intra-Cultural Universality:** … entails that (almost) all persons within P’s culture have a basic need for x (e.g., Brock, 2005; Reader, 2007).
(F10) **Cross-Cultural Universality:** … entails that (almost) all persons across (almost) all cultures have a basic need for x (e.g., Brock, 2005; Reader, 2007).
(F11) **Fallibility:** … entails that P as well as other individuals and collectives can be mistaken about whether P has this basic need (Doyal & Gough, 1991; Pinzani, 2013; Reader, 2007; Thomson, 2005).
(F12) **Objectivity (Individual):** … entails that whether P has a basic need for x is independent from whether P believes that he/she has this basic need (Gough, 2015; Pinzani, 2013; Reader, 2007; Thomson, 2005).
(F13) **Objectivity (Culture):** … entails that whether P has basic need for x is independent from whether P’s culture dominantly believes that he/she has this basic need (Gough, 2015; Pinzani, 2013; Reader, 2007; Thomson, 2005).

Our first two studies were meant to explore the extent to which ordinary speakers endorse F1 to F13. We will now turn to presenting and discussing these studies.

## Study 1: hypothetical cases

Study 1 proceeded by presenting participants with hypothetical cases that each fail to exhibit one potential feature of *basic needs*. Then they were asked whether they believe that the concept could still apply. This method mimics philosophers’ “method of cases” (Machery, 2017; Williamson, 2007), as it is commonly used in analyzing concepts, and as it has also been employed in other experimental philosophy research (e.g. Horvath & Wiegmann, 2016; Swain et al., 2008).

### Participants

We recruited 166 participants via Prolific Academic, an online crowdsourcing platform. As inclusion criteria, we required that participants (1) reside in the US or UK, (2) speak English as their first language, and (3) have completed anywhere between 10 and 200 studies with (4) an approval rate no lower than 90%. Seven participants were excluded from analysis because they failed the attention checks in our survey, and a further six were excluded for completing the study in less than four minutes. Among the remaining 149 participants, ages ranged from 20 to 60 (*M* = 28.89, *SD* = 7.53), and 79.5% were women. Further demographic information is provided in Appendix A.

### Methods

Participants first received instructions about the study’s aim and method. For each of F1 to F13 they were then presented with a hypothetical case in which a person’s putative basic need for an unspecified thing X fails to exhibit this feature. They were asked whether the person could nevertheless be said to have a basic need. For example, the scenario for *Harm-Avoidance* looked as follows:

*Harm-Avoidance*

Suppose a person completely lacks X. As a result, the person is not harmed in any serious way. The person remains mostly unharmed. There are hardly any negative consequences for their life or functioning. Now consider the following statement: “It could still be appropriate to say that this person has a basic need for X even though the lack of X does not harm them in any serious way.”

Participants were asked to rate their agreement with the statement at the end of each scenario on a scale ranging from 1 = “strongly agree” to 7 = “strongly disagree”. According to our interpretation, the more participants disagreed with this statement the more strongly they endorsed the respective feature as necessary for, or characteristic of, *basic needs*.^5^ For example, if a participant disagrees that a person could be said to have a basic need for X even though the person is not harmed in any serious way by lacking X, they are thereby expressing their agreement with the idea that the absence of X must result in serious harm to a person for that person to have a basic need for X. This amounts to an endorsement of the principle that basic needs are defined as serving the goal of harm-avoidance.

After these scenarios participants were asked some additional questions and had to provide basic demographic information.

### Results

A one-way ANOVA with feature as a within-subjects factor revealed significant variation in agreement across features, *F*_(13, 1924)_ = 17.81, *p* < 0.001. We conducted signed-rank tests against the scale midpoint to examine whether, in the aggregate, participants endorsed or rejected each of F1 to F13 (see Appendix B).

**Fig. 1:**
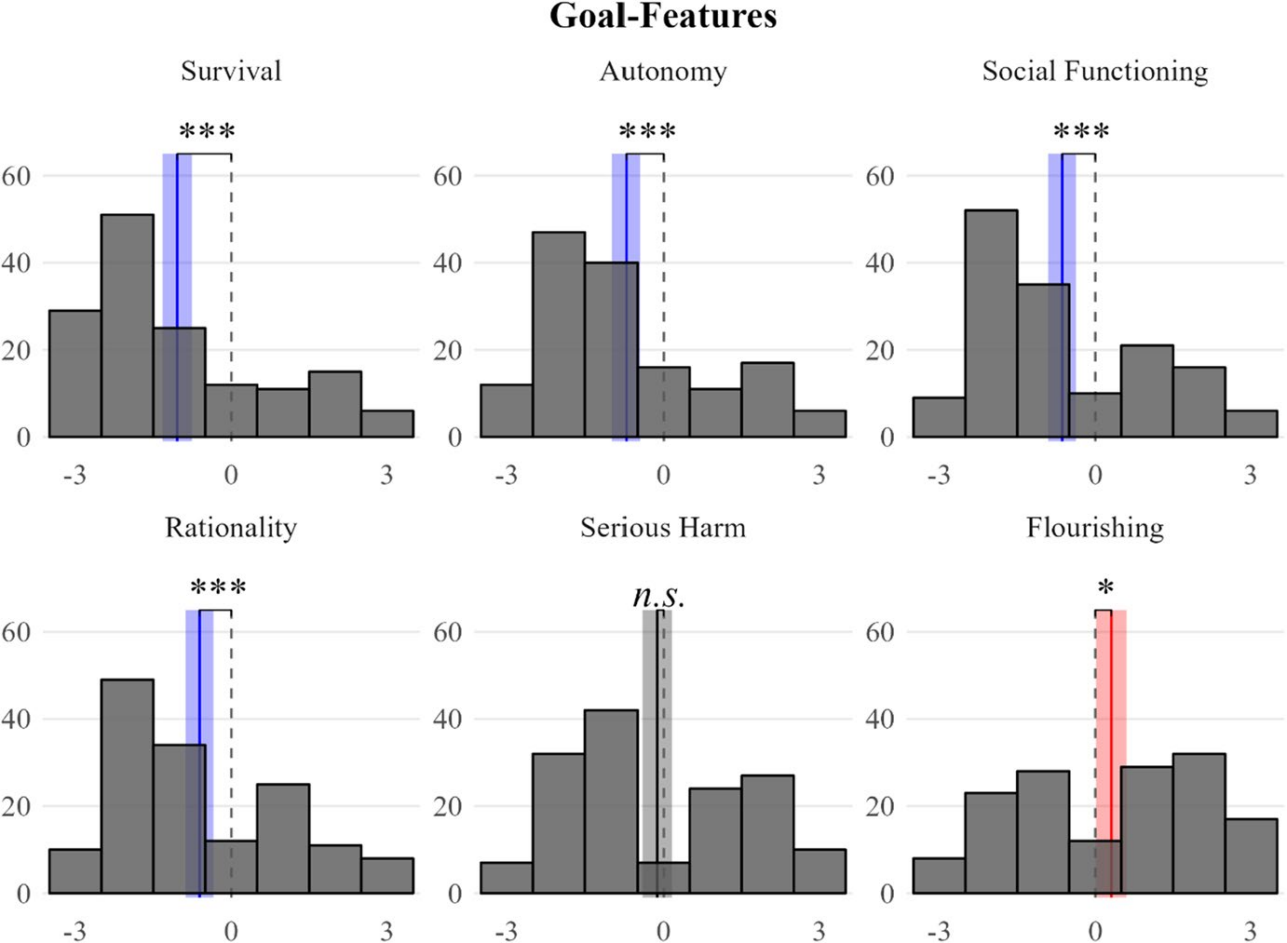
Histogram of endorsement of F1 to F6 (as explained in Sect. 3), with the degree of endorsement on the x-axis (from  − 3: rejection to + 3: endorsement), and the number of responses on the y-axis. The red vertical lines represent the sample means and the surrounding shaded band spans the corresponding 95% confidence interval. Non-overlap between the 95% confidence interval and the scale midpoint (dashed grey) indicates a statistically significant difference: *p < 0.05, **p < 0.01, ***p < 0.001

Endorsement of the six features that specify goals of basic needs satisfaction (F1 to F6) varied. Participants denied that basic needs must enable recipients to survive, act rationally, act autonomously, or function socially, all *p*s < 0.001. By comparison, they were more favorable towards the two remaining goal features. The question of whether basic needs must lead to the avoidance of serious harm prompted divided responses (*M* =  − 0.13, *p* = 0.480), and participants agreed that for a thing to be a basic need it must help its recipient flourish in the sense of living “well and happily” or “growing as a person” (*M* = 0.31, *p* = 0.036) (see Fig. 1).

**Fig. 2:**
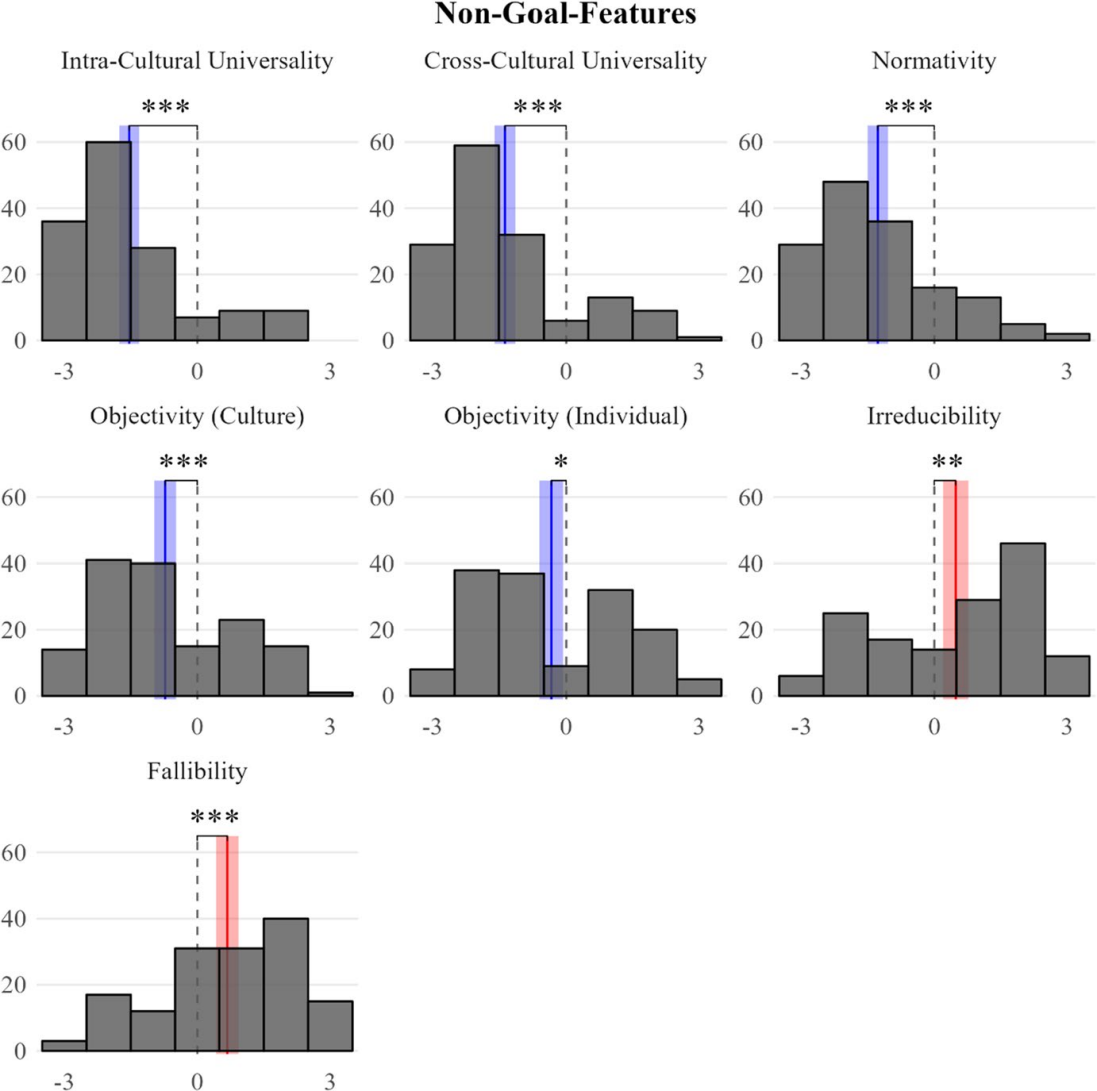
Histogram of endorsement of F7 to F13 (as explained in Sect. 3), with the degree of endorsement on the x-axis (from  − 3: rejection to + 3: endorsement), and the number of responses on the y-axis. The red vertical lines represent the sample means and the surrounding shaded band spans the corresponding 95% confidence interval. Non-overlap between the 95% confidence interval and the scale midpoint (dashed grey) indicates a statistically significant difference: *p < 0.05, **p < 0.01, ***p < 0.001

Among the concept’s potential non-goal features (F7 to F13), participants most strongly denied the universality of basic needs (*M*s =  − 1.36 (intra),  − 1.54 (cross), *p*s < 0.001). To a lesser extent, they also denied that these needs are objective (*M* =  − 0.34, *p* = 0.012 (individual), *M* =  − 0.73, *p* < 0.001 (cultural)) and that they impose obligations on people (*M* =  − 1.28, *p* < 0.001). The only two non-goal features that were endorsed are fallibility (*M* = 0.68, *p* < 0.001) and irreducibility (*M* = 0.48, *p* = 0.002); i.e., participants indicated that, in their view, people can be mistaken about their basic needs and these needs are not just a sub-category of preferences (see Fig. 2).

### Discussion

The three most surprising findings of Study 1 pertain to the breadth of the folk concept of basic needs, to its objectivity and to its universality.

Most normative theorists have defined basic needs in a narrow sense, according to which *x* is a basic need if and only if having/being/realizing/etc. *x* is required to avoid (serious) harm (such as harm that consists in impairments of autonomy, rational agency, or social functioning; e.g., Copp, 1995, 1998; Doyal & Gough, 1991; Wiggins, 1998). The participants in our study, in contrast, were divided about the goal of harm-avoidance. Moreover, many participants also seem to recognize broader usages of the concept. They were disposed to classify not only what is required for a minimally decent life as a basic need, but also what is required for flourishing. This is reflected by the relatively high endorsement of the flourishing-feature, which ranked highest among the goal features and exceeded the serious harm-feature.

Perhaps even more surprising is that participants in Study 1 strongly denied that basic needs must be objective and, in particular, universal. One potential worry about this finding is that participants may have confused basic needs (such as food) with the means of these needs’ satisfaction (such as Wiener Schnitzel or Sushi; for this distinction see Max-Neef, 1991). More specifically, in thinking about the “X” in our scenarios they may have only or primarily had these means in mind — which even proponents of needs-based normative theories regard as subjective and relative (e.g., Brock, 2005; Doyal & Gough, 1991).

However, this explanation likely does not apply to many participants. After providing their above ratings participants of Study 1 were asked the following open-ended question: “When completing the tasks, did you think of any particular basic need/s (that you put in place of ‘X’)?”. In response to this question the majority of answers did not so much refer to means of satisfaction but rather, as intended, to the more abstract level of basic needs themselves. Representative answers include “Food, water, shelter, clothes” and “I tried not to think of specific basic needs. At times things like ‘food’ or ‘water’ or ‘love’ came to mind, but for the most part they did not.”^6^

According to another potential worry, the concept of basic needs is not part of ordinary English discourse at all—which means that ordinary speakers’ intuitions do not reflect competency in applying the concept and are hence irrelevant to justifying analyses of *basic needs* (see, e.g., Kauppinen, 2007; Ludwig, 2007, 2010). Our additional questions to some extent answer to this worry as well. Participants indicated that they are more familiar than not with the concept, 2.72 on a scale from 1 = “extremely familiar” to 5 = “not familiar at all”. When asked about the contexts in which they have used or encountered it, they provided answers such as “I am studying occupational therapy and we talk a lot about needs, basic and otherwise”, “Being isolated during COVID. Showed how much human interaction was important to me and how much interaction I needed”, and “I think of basic needs when paying bills.”^7^

Study 1 may also be criticized for its operationalization of harm-avoidance analyses (which typically involve a narrow understanding of *basic needs*). In particular, we only tested the following version of such analyses: P’s having a basic need for x entails that if P does not have/realize/etc. x then P is *seriously* harmed (see Sect. 3). Endorsement ratings would likely have been higher, critics might argue, if we had instead investigated intuitions about the necessity of harm in an unqualified sense, i.e., of serious or non-serious harm. This has led us to underestimate the support for narrow analyses of *basic needs*.^8^

Normative theorists have so far mostly considered *serious* harm as a necessary condition of basic needs. For example, Thomson states that “‘A has a need for X implies that X is practically necessary specifically for A […] when he cannot do without it, when his life will be blighted or seriously harmed without it’” (1987: 8; see also, e.g., Doyal & Gough, 1991). Still, we decided to conduct a small follow-up study to test the above prediction. This study (*N* = 153) involved (slightly simplified) versions of the serious harm and flourishing tasks, as described in Sect. 4.2, and a test of the endorsement of a new unqualified harm feature: “It could still be appropriate to say that this person has a basic need for X even though the lack of X does not harm them.”

We found that both the new unqualified harm feature and the serious harm feature were rejected in comparison with the theoretical midpoint of the scale (*M*_*harm*_ =  − 0.32, *p* = 0.020; *M*_*serious*_ =  − 0.84, *p* < 0.001) and, as predicted, the harm feature was rejected less strongly than the serious harm feature (*t*(302) = 3.74, Cohen’s *d* = 0.43, *p* < 0.001). However, the difference was rather small and does not affect our overall interpretation of Study 1 in any way.^9^ Participants were again more likely to agree that for a thing to be a basic need it must help its recipient flourish (*M* =  − 0.11, *p* = 0.510) rather than avoid serious harm (*t*(302) = 5.24, Cohen’s *d* = 0.60, *p* < 0.001), and the flourishing feature was even endorsed more strongly than the principle that basic needs require the avoidance of any kind of harm (*t*(302) = 1.50, Cohen’s *d* = 0.17, *p* = 0.140), although the difference was non-significant.

In our view, the most serious worry about Study 1 is more general. To fully understand our scenarios and questions participants had to engage in complex counterfactual thinking about an entity that was only described abstractly (“X”) to access their modal (rather than actual) intuitions. Some or many participants may have struggled with this cognitively complex task. This worry might also be supported by the fact that our data showed only modest variation across features (see Figs. 1 and 2). Hence, we decided to conduct a follow-up study on the same thirteen potential features of *basic needs* that employed a simpler design.

## Study 2: statements

Study 2 presented participants with two short statements for each feature. The first statement endorsed the feature; the second one denied it. We also manipulated whether the features were stated as basic needs’ necessary properties or as empirical generalizations (analogously to previous experimental philosophy research, see Donelson & Hannikainen, 2020).

### Participants

Based on the same prescreening criteria as in Study 1, we recruited 318 participants via Prolific Academic. We excluded four participants from further analyses who failed our attention checks or provided incomplete responses. This resulted in a sample of 314 participants, whose ages ranged between 17 and 70 (*M* = 30.91, *SD* = 8.83), and 69.1% of whom were women. Further demographic information is provided in Appendix A.

### Methods

Study 2 tested the degree of endorsement of the same 13 features as in Study 1, i.e., F1 to F13. Participants were randomly assigned to either an actual or modal condition. In the actual condition, they were presented pairs of statements that were formulated in terms of what basic needs are actually like, for example:

*Universality (Culture)*

Basic needs differ from one culture to the next.

Basic needs do not differ from one culture to the next.

In the modal condition, the statements were formulated in terms of what basic needs *could* or *must* be like, for example:

*Universality (Culture)*

Basic needs could differ from one culture to the next.

Basic needs could not differ from one culture to the next.

For each pair of statements, participants had to pick the statement that better reflected their view. Then they were asked an additional question about their interpretation of the task; in particular, about whether they were thinking about (1) “what basic needs are usually like, in your experience”, (2) “what basic needs must be like, as in the requirements for something to count as a basic need”, or (3) “what basic needs should be like, according to your beliefs about right and wrong.”

### Results

**Fig. 3:**
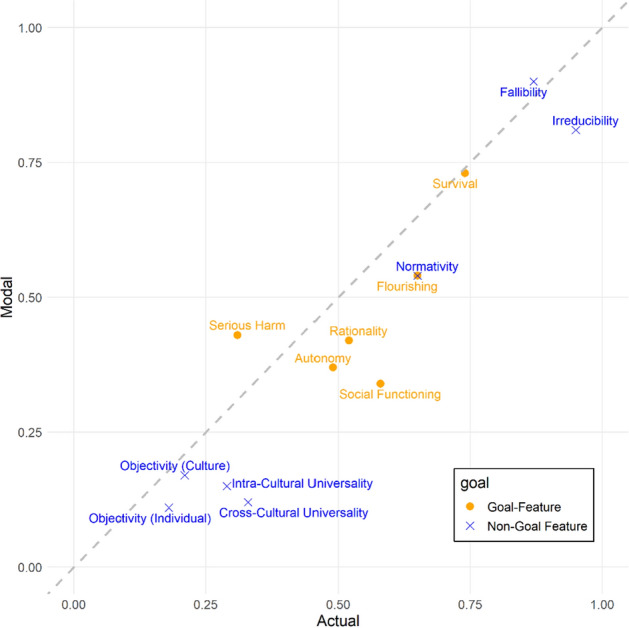
Scatter plot of endorsement of F1 to F13 (as explained in Sect. 3) in the actual (x-axis) and modal (y-axis) conditions

A series of Wilcoxon rank sum tests showed that participants endorsed the serious harm feature more strongly in the modal than in the actual condition (*W* = 10,710, *p* = 0.022); all other features were endorsed more strongly in the actual condition (*W*s > 13,623, *p*s <  0.043) or equally strongly in both conditions (*W*s < 13,456, *p*s > 0.080) (see Fig. 3). Nonetheless, the rates of endorsement were strongly correlated across conditions, Spearman’s *rho* = 0.89, *p* < 0.001. In what follows we will therefore focus on the modal condition, which is the one that is most relevant to conceptual analyses (for the results of the actual condition see Appendix B).

We conducted a series of one-way proportion tests against the null hypothesis that the proportion equals 0.50 to examine whether endorsement of each feature was significantly different from chance. Among the goal features, flourishing again ranked highly, although this time the result did not reach the level of statistical significance (*Prop.* = 0.54, *p* = 0.430). The only feature that surpassed flourishing was survival. Participants widely endorsed the statement “Meeting basic needs must ultimately always help us to survive” (*Prop.* = 0.73, *p* < 0.001). In contrast, participants tended to reject the claim that basic needs must help recipients function socially (*Prop.* = 0.34, *p* < 0.001), and be autonomous (*Prop.* = 0.37, *p* = 0.001), and were divided as to whether they must help recipients act rationally (*Prop.* = 0.42, *p* = 0.055) and must preclude serious harm (*Prop.* = 0.43, *p* = 0.11).

As to the non-goal features, participants again strongly denied that basic needs are objective (*Prop*s. = 0.17 (individual), 0.11 (cultural), *p*s < 0.001), and universal (*Prop*s = 0.15 (intra), 0.12 (cross), *p*s < 0.001). The non-goal features that garnered most agreement were fallibility and irreducibility. That is, most participants indicated that “[p]eople could sometimes be mistaken about what their basic needs are” (*Prop* = 0.90, *p* < 0.001) and that “[b]asic needs must be more than something that we just want” (*Prop* = 0.81, *p* < 0.001). We also found slight, though statistically non-significant, endorsement of the claim that basic needs impose obligations on others to enable their fulfillment (*Prop* = 0.54, *p* = 0.425).

Finally, our interpretation question revealed that in both conditions, participants were more likely to interpret the presented statements as normative claims (what basic needs should be like) than as modal (what they necessarily must be like) or empirical (what they are actually like) claims, *F* (2, 622) = 29.97, *p* < 0.001, normative vs. actual: *t* (312) = 6.88, *p* < 0.001; normative vs. modal: *t* (312) = 2.82, *p* = 0.005), actual vs. modal: *t* (312) = 5.09, *p* = 0.005.

### Discussion

Study 2 to a large extent replicated the three main findings of Study 1, regarding the breadth of the folk concept of basic needs, its objectivity and its universality.

Participants’ responses indicate that they recognize narrow elements of the concept. The highest ranked goal feature was survival, which is even narrower than harm-avoidance, the most widely endorsed narrow feature in Study 1 (survival does not even guarantee a minimally decent life; just life as such). However, many participants were again disposed to allow for broader usages as well. They in particular again showed relatively high endorsement of flourishing, as represented by the statement “Meeting basic needs must ultimately always help us grow as people and/or live well” (second highest ranked goal-feature). This result is consistent with Study 1, even though this time we cannot rule out that it came about by chance.

Just like with Study 1 as well, our perhaps most striking and clearest finding was that participants widely rejected basic needs’ objectivity (ranks ten and thirteen) and universality (ranks eleven and twelve). In the case of objectivity this finding might be questioned by pointing to a *prima facie* tension with the high endorsement of fallibility. How can one be mistaken about a fact (fallibility) if what grounds this fact are one’s own beliefs about it (individual subjectivism)? By analogy, if I hold that vanilla ice cream tastes good if and only if I believe that vanilla ice cream tastes good then it seems contradictory to also hold that I can have false beliefs about whether vanilla ice cream tastes good.

At this point we cannot explain participants’ simultaneous rejection of individual objectivity and their endorsement of fallibility. The most plausible explanation, in our view, is that they endorsed both features in a qualified sense. In particular, participants may have held that even though basic needs depend on our beliefs, this does not hold for *all* of them, or not *fully*. That said, it is also possible that ordinary speakers really do hold (somewhat) contradictory beliefs when it comes to basic needs’ objectivity and fallibility. It would then be the task of normative theorists to resolve these contradictions in their analyses, thereby departing from the folk concept (see Sect. 2).

One worry about Study 1 was that participants may have interpreted the presented materials as asking about what basic needs are *actually* like. Our additional question suggests that in fact, they may dominantly think about what these needs *should* be like, “according to your beliefs about right and wrong”. To the extent to which “right” and “wrong” were interpreted as linguistic/theoretical rightness and wrongness, this is fully consistent with our studies’ aim of probing conceptual intuitions. An interpretation in terms of moral/practical rightness and wrongness, in contrast, would further support that people consider the concept of basic needs to be inherently practically normative.

In any case, the fact that participants in the modal condition did not primarily report a modal interpretation of the task (namely that they are about “the requirements for something to count as a basic need”) may raise concerns about the internal validity of Study 2. We therefore decided to approach ordinary speakers’ intuitions from yet another methodological angle.

## Study 3: qualitative data

Philosophers who deny that scientific studies can contribute to determining ordinary speakers’ conceptual intuitions typically object to the relevance of quantitative research (e.g., Bengson, 2013; Kauppinen, 2007). There is also another potential problem with the approach that we have taken in Studies 1 and 2. These studies only asked participants about potentially necessary or characteristic features that have been previously raised in the academic literature. It is possible, however, that ordinary speakers’ usage of the concept of basic needs is in fact determined by features that philosophers and social scientists have entirely neglected. For these reasons we decided to gather qualitative evidence about folk intuitions on the concept of basic needs (in line with suggestions by, e.g., Andow, 2016).

Some research of this kind has already been reported in a previous paper (see Pölzler & Hannikainen, 2022). There participants were asked the following question: “What are basic needs? What comes to mind when you think about this concept? […]” The most noteworthy feature of the results was that all of this study’s participants (95 out of 95) provided examples of the concept of basic needs, such as “water”, “food” or “shelter”. No participant associated any abstract feature with the concept, such as “necessary to prevent serious harm” or “necessary to be function socially”.

We have argued that this result suggests a non-classical structure of the concept. In deciding whether *basic needs* applies to an item people do not check whether the item fulfills a set of necessary and sufficient conditions. Instead, they may compare the item to a stored prototype that was formed by abstracting certain characteristics, as well as the relative statistical prominence of these characteristics, from the most typical instances of the concept (Hampton, 2006; Rosch, 1973, 1975, 1978; Rosch & Mervis, 1975). This means that not all instances of *basic needs* might share the same features; rather, as Wittgenstein (1973) remarked with regard to the concept of games, we might see a “complicated network of similarities overlapping and criss-crossing” (PI §66). For example, water might be categorized as a basic need because it shares features a, b, e, while education might be categorized as a basic need because it shares features b, d, f.^10^

**Fig. 4:**
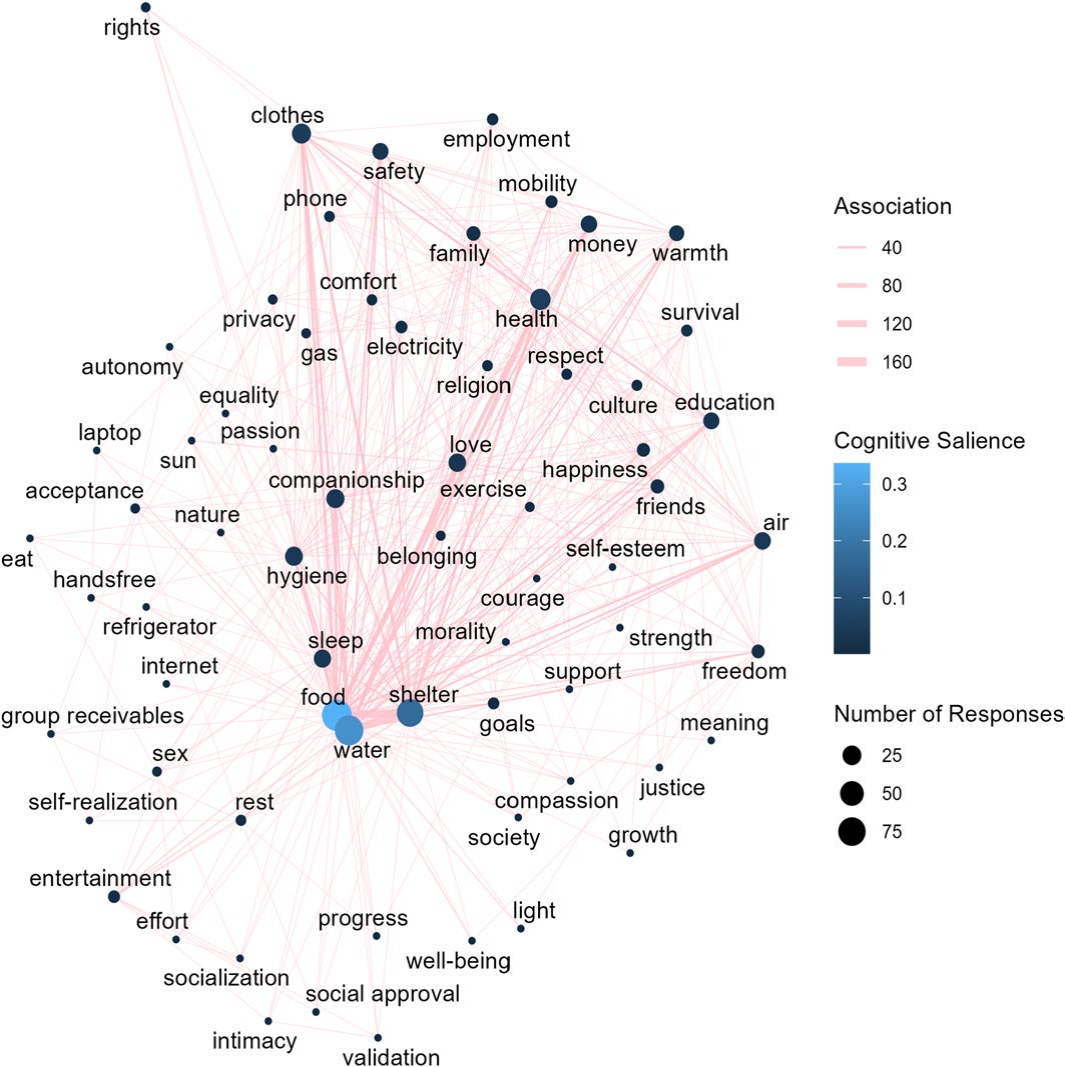
Network graph showing interconnections between the items listed in BLINDED, with the size of dots representing the frequency with which the item appeared, the width of lines representing the frequency with which two items appeared in the same participant’s list, and the color of dots representing cognitive salience. The cognitive salience of each item was calculated on the basis of a formula devised by Sutrop (2001): frequency/(sample size * mean position)

Another relevant finding of this related research was that participants most often and earliest associated items with *basic needs* that are related to survival and harm-avoidance, in particular food (mentioned by 95, i.e., all participants), water (84) and shelter (69). This may be claimed to support a narrow understanding of the concept. However, evidence of the broader understanding, as documented in Studies 1 and 2, emerged as well. For example, 22 participants associated “companionship” with the concept, 20 participants associated “love”, 14 participants associated “education”, and 7 participants associated “happiness”. In Fig. 4, we re-analyze the results of this study (Study 1 in Pölzler & Hannikainen, 2022), displaying the relationships between different items.

One limitation of the aforementioned qualitative data is that it only provides indirect evidence of the necessary or characteristic features attributed to the concept of basic needs (evidence about the concept’s mental structure and the items that might be involved in forming prototypes). For another task we hence decided to take a more direct approach.

### Participants

Based on the same prescreening criteria as in Study 1, we recruited 100 participants via Prolific Academic. Seven participants were excluded from analysis because they finished the survey in less than two minutes. Of the remaining 93 participants, ages ranged from 19 to 72 (*M* = 30.1, *SD* = 11.5), and 39% were women. Further demographic information is provided in Appendix A.

### Methods

The materials of Study 3, which were presented to participants after they had completed the above-mentioned research in Pölzler & Hannikainen (2022), directly asked them to explain their understanding of the concept in a more abstract sense:

How would you explain the concept of basic needs to a person who is unfamiliar with it? Please provide a more coherent and extensive explanation of what basic needs mean to you. For example, you may find it useful to state why something counts as a basic need, the different types of basic needs there are, or the qualities that basic needs typically share.

Having completed this task, participants were also asked a series of demographic questions.

### Results

**Fig. 5:**
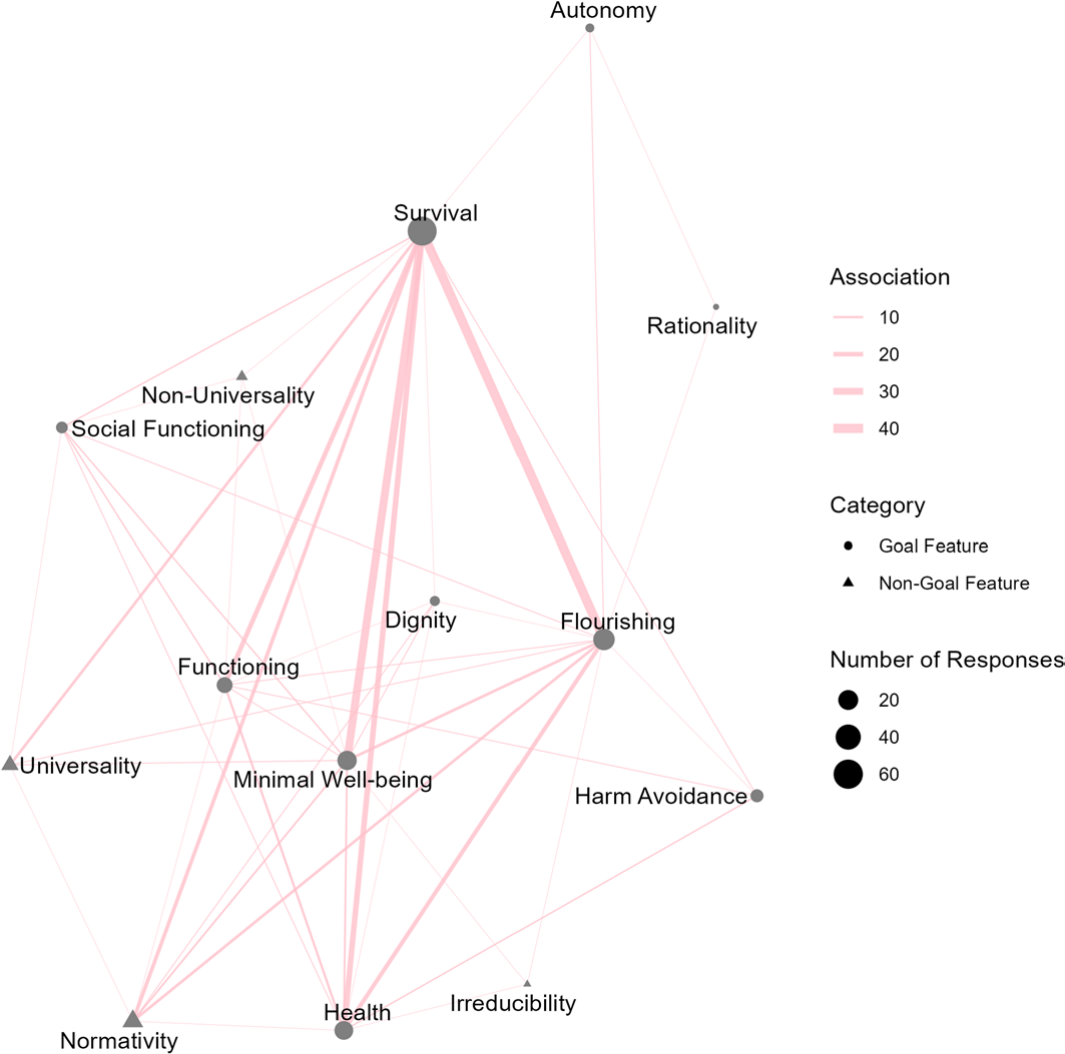
Network graph showing interconnections between the features listed in Study 3, with the size of symbols representing the frequency with which the feature appeared, the shape of symbols representing the type of features, and the width of lines representing the frequency with which two features appeared in the same participant’s list

Two coders independently interpreted the responses, with disagreements being resolved by the first author in consultation with the third author. Intercoder reliability checks revealed Gwet’s AC1 indices of > 0.60 for all items, with an average of 0.86 (Byrt et al., 1993; Gwet, 2014). Results revealed that ordinary speakers often define *basic needs* in terms of the same features that have been discussed by normative theorists. The only five additional features that were listed are mostly goal-related. In particular, some participants explained that basic needs are those things that are required to achieve (1) functioning, (2) health, (3) dignity or (4) minimal well-being. In addition, a small number of participants provided explanations in terms of (5) non-universality. Table 1 provides summary statistics of the frequency of each feature resulting from our coding of the data. Figure 5 depicts the extent to which participants combined different features in their definitions.

**Table 1:** Frequency of all features that were mentioned in Study 3, with “*” indicating features that were added beyond F1 to F13

Feature	Frequency
Survival	67
Flourishing	31
Minimal Wellbeing*	23
Health*	21
Functioning*	13
Normativity	12
Harm-Avoidance	7
Universality	7
Social Functioning	5
Dignity	3
Autonomy	2
Non-Universality*	2
Rationality	1
Irreducibility	1

### Discussion

The results of Study 3 indicate that many ordinary speakers’ first and foremost understand *basic needs* in a narrow sense that most strongly relates to survival. However, once again, the results provide evidence for a broader, more inclusive understanding of the concept as well. Flourishing (in the sense of either having a good/happy life or growing as a person) was the second most often cited feature. Moreover, 74.6% of those participants who defined basic needs in terms of survival did not *only* define it in these terms. They also mentioned some of the other features, most often flourishing or minimal well-being (see Fig. 5), which renders their concepts broader than the elevated endorsement of survival might initially suggest.

Participants’ responses are also consistent with our previous findings about ordinary speakers’ rejection of the objectivity and universality features. No participants in Study 3 mentioned anything vaguely resembling objectivity in attempting to define *basic needs*. Moreover, to the extent that participants *did* make claims to universality, these claims mostly pertained to the existence of basic needs (all humans have basic needs) or to these needs’ normativity (all humans have a right to basic need satisfaction) — neither of which entails that all humans have the *same* basic needs. Two participants even explicitly denied that basic needs are universal.

## Implications for analyses

In the previous sections, we presented three studies that examined the extent to which ordinary English speakers intuitively endorse a series of features of the concept of basic needs. We will now move on to discussing the potential implications of these findings for philosophers’ attempts to analyze this concept.

### Breadth

Normative theorists have typically assumed a narrow understanding of *basic needs*. As mentioned before, most of them have defined these needs as necessary for avoiding serious harm (e.g., Brock, 2005; Copp, 1995; Doyal & Gough, 1991; Miller, 1999). On this definition people exclusively or almost exclusively have basic needs that are *physiological* in kind — i.e., for things like food, water, shelter, clothes, and other fundamental goods.

In some theoretical contexts a narrow conception of *basic needs* makes sense; for example, when it comes to defining a threshold of minimal well-being (e.g., Meyer & Pölzler, 2022; Miller, 1999). We hence do not mean to question the legitimacy of singling out this prototypical core of the folk concept (see Sect. 2). At the same time, our results show that at least in terms of conformity with ordinary speakers’ usage, a broader analysis can be justified as well. Exploring the fruitfulness of such an analysis could be philosophically worthwhile.

To our knowledge, the only researcher who has so far seriously considered defining basic needs in terms of flourishing (beyond a merely unharmed, minimally good life) is Stewart (1985).^11^ Stewart stipulated that a person has a basic need for *x* if and only if *x* contributes to a full life. For pragmatic reasons, she decided to interpret the notion of a full life in a narrow sense. But she pointed out that a broader interpretation could be equally legitimate.

[…] the BN-objectives [are] the improvement of conditions of life (or quality of life). The bundle of BN-goods are then selected according to whether or not they contribute to this ultimate objective - which for shorthand we describe as the ‘full-life objective’. The full-life objective may be defined extensively or minimally. A minimal definition confines the objective to health and perhaps education. An extensive definition would include all sorts of other characteristics such as conditions necessary for the enjoyment of art, for entertainment generally, for full participation in the political process, and so on. (Stewart, 1985: 3)

Stewart’s extensive definition has typically been dismissed as being too broad (see, e.g., Gaspar, 1996; Schuppert, 2013). Yet, our studies suggest that this might actually not be the case—at least not in terms of fit with the concept’s common understanding.

Defining basic needs in a broad sense might also be supported by independent theoretical reasons. In particular, it could allow proponents of needs-based normative theories to reap advantages which have so far been considered reserved to those who have appealed to basic capabilities (Nussbaum, 2006). For example, Fardell (2020) has argued that the concept of basic needs might be defined in a way that includes basic needs for freedoms to do certain things. This would help counter worries about needs-based theories being paternalistic (see below).

The main challenge for broader analyses of *basic needs* is to preserve the concept’s independent normative relevance, i.e., to show that even basic needs *qua* necessities for a flourishing life ground *pro tanto* reasons for their satisfaction. On a broad analysis the concept also might not have as much motivational and rhetorical force as on a narrow one (Schuppert, 2013). Future empirical studies could help assessing these potential disadvantages by manipulating ordinary speakers’ understanding of the concept (broad versus narrow) and then investigating how these manipulations affect participants’ willingness to attribute normativity to basic needs and their motivation to enable others to meet these needs.^12^

### Subjectivity

A large majority of philosophers have defined basic needs as objective, i.e., as independent from what individuals and cultures think about them (e.g., Gough, 2015; Pinzani, 2013; Reader, 2007; Thomson, 2005).

This supposed objectivity has been one of the main attractions of the concept in normative contexts. Among other things, on the basis of objectivist analyses, it has been claimed that basic needs and their satisfaction can be more easily and reliably assessed than on the basis of non-objective ones (e.g., states can to a large extent rely on well-established and widely applied social indicators such as life expectancy at birth, health expenditure or poverty rates; Reader, 2007); and that such analyses shield needs-based normative theories from the so called adaptive preference problem^13^ (even if some people might not *prefer* the goods that are necessary to satisfy their basic needs the fact that they do not have access to these goods would still allow us to regard them as being badly off; Gough, 2015; Page, 2007).

Our finding that most ordinary speakers deny the objectivity of basic needs puts some *prima facie* pressure on needs theorists to drop this feature. One way to avoid doing so would be to diverge from the folk concept in this respect. This might be supported by the above-mentioned finding that ordinary speakers endorse fallibility which, as said, logically entails objectivity (see Sect. 5). However, the plausibility of this reply is doubtful. Our results suggest a relatively strong subjectivist tendency in ordinary discourse on basic needs. Moreover, this tendency accords with people’s intuitions about other normative concepts. For example, research on folk moral objectivism has recently converged on the finding that most of the time laypeople regard moral statements as non-objective (e.g., Davis, 2021; Pölzler & Wright, 2020; Sarkissian et al., 2011).^14^

Alternatively, proponents of needs-based normative theories may reconsider their strongly objectivist position. There are several ways of doing so that are consistent with our data. Among other things, it might be argued that only some small set of basic needs are objective, while the majority of them are subjective; or that, even though objectivity is not a necessary or strongly characteristic feature of (all) things that we categorize as basic needs, it is at least weakly characteristic (in the sense that objectivity brings an item at least somewhat closer to the threshold that it must meet to be categorized as a basic need).

Allowing room for at least some amount of subjectivity might again be supported by independent reasons as well. Full objectivity comes with some advantages, as described above, but also with one major disadvantage. On objectivist needs-based theories, individuals and cultures have no real say in what their basic needs are. This has often inspired allegations of paternalism, i.e., disrespect for recipients’ autonomy (e.g., Alkire, 2002; Sen, 1984). By claiming only partial objectivity, needs-based normative theories may become more philosophically appealing.

### Relativity

Similar considerations apply to ordinary speakers’ endorsement of the relativity of basic needs. The standard analysis put forth by normative researchers treats basic needs as universal. That is, it entails that all (or almost all) individuals within and even across cultures have the same basic needs (e.g., Brock, 2005; Reader, 2007).

Just like with objectivity, basic needs’ alleged universality has been claimed to constitute a theoretical advantage (e.g., Brock, 2005; Meyer & Pölzler, 2022). Sometimes the well-being related attitudes and practices of particular populations cannot be studied, at least not directly. For example, it may not be safe to study populations in conflict-ridden parts of the world; and we cannot possibly study people in the far-off future, as they have not even yet been born. This means that we do not know and often cannot even reliably predict what these people prefer, what capabilities they value, etc. But if basic needs were universal then we could nevertheless know what they *need*—by simply extrapolating from our own basic needs.

To preserve this epistemic advantage, the initial impulse of needs-theorists might again be to revise or override folk intuitions. But more conciliatory replies may again be plausible too. Just as in the case of objectivity, participants’ rejection of full universality was strongly in line with a broader research program in moral psychology, which suggests that people regularly relativize moral statements to particular individuals or cultures (e.g., Kelly et al., 2007; Quintelier et al., 2013). Thus, in some theoretical contexts, needs theorists might instead want to acknowledge some degree of relativity. This can still be compatible with extrapolating basic needs across individuals or cultures sometimes or to some extent.

## Conclusion

In this paper we have attempted to inform discussions about the meaning of the concept of basic needs by gathering empirical evidence about ordinary English speakers’ conceptual intuitions. In light of our findings’ convergence across different approaches, we take it that overall our research provides evidence for the following claims: (1) ordinary speakers sometimes apply the concept of basic needs to necessities for a flourishing (not just a minimally decent) life, (2) most ordinary speakers attribute at least some degree of subjectivity to the concept, and (3) most ordinary speakers attribute at least some degree of relativity to the concept.

Two qualifications are in order. First, in this paper we have only discussed how English speakers use the English term “basic needs”. To what extent the corresponding concept in *other* languages is to be understood in a broad, subjectivist and relativist sense is yet unknown. For example, can we really assume that the Indonesian term “kebutuhan pokok” or the Japanese term “基本的ニーズ” share all the same necessary or characteristic features as “basic needs” does among English speakers? Echoing previous evidence that people from WEIRD (i..e, Western, educated, industrialized, rich and democratic) societies often have a peculiar set of intuitions (Henrich et al., 2010), we caution against making such generalizations—particularly to *non*-WEIRD linguistic communities.

This is a serious limitation. The concept of basic needs is sometimes drawn on in precisely those contexts in which the bearers and recipients of obligations are dispersed across cultures—such as when theorists ask what North American or European governments owe people in developing countries as a matter of global justice (e.g., Brock, 2009; Doyal & Gough, 1991). Future research should hence explore the features that guide ordinary speakers’ application of the concept of basic needs in several languages besides English. In fact, we have recently already started such cross-cultural follow-up studies (Pölzler et al., under review).^15^

Second, we would like to remind our readers that we do not mean to suggest that our findings should fully or even largely determine the outcome of analyses of the English term *basic needs* either (see Sect. 2). Our findings still leave plenty of room for revision and adaptation to different theoretical contexts. We only take our investigations to suggest that normative theorists should at least *take seriously* analyses of *basic needs* that are broader than those that have been advanced through the stipulative approach, and that are (at least partially) subjectivist and relativist. This might still amount to an important reconsideration of what it means for people to have basic needs. In particular, the minimum that each person is owed may be less minimal and less rigid than normative theorists have thought.

## Appendix A: participants

### Study 1

We recruited 166 participants via Prolific Academic. 10 participants were excluded because they failed attention checks. 7 participants were excluded because they completed the survey too fast. Of the remaining 149 participants, 31 identified as men and 118 identified as women. The mean age was 29.9 years (*SD* = 8.66). Participants were generally politically left leaning (*M* = 2.97, *SD* = 1.45, where 1 = ‘Liberal’ and 7 = ‘Conservative’), economically slightly left leaning (*M* = 3.45, *SD* = 1.71, where 1 = ‘Liberal’ and 7 = ‘Conservative’) and socially left-leaning (*M* = 2.69, *SD* = 1.51, where 1 = ‘Liberal’ and 7 = ‘Conservative’). They were primarily Caucasian (*n* = 118, or 79.19%) and largely non-religious (*n* = 74, or 49.66%), as opposed to weakly (21.48%), moderately (20.81%) or strongly (7.38%) religious. The majority of participants (*n* = 119, or 79.87%) reported a yearly income of less than USD 70,000, and a sizeable minority (*n* = 52, or 34.90%) reported a yearly income of less than USD 30,000.

### Study 2

We recruited 318 participants via Prolific Academic. 3 participants were excluded because they failed attention checks. 1 participant was excluded because they failed to provide complete data. Of the remaining 314 participants, 86 identified as men and 217 identified as women, with 2 participants who preferred not to say and 9 not providing any response at all. The mean age was 30.91 years (*SD* = 8.83). Participants were generally politically centrist (*M* = 3.96,* SD* = 1.77, where 1 = ‘Liberal’ and 7 = ‘Conservative’), economically slightly right leaning (*M* = 4.31, *SD* = 1.76, where 1 = ‘Liberal’ and 7 = ‘Conservative’) and socially slightly left-leaning (*M* = 3.63, *SD *= 1.85, where 1 = ‘Liberal’ and 7 = ‘Conservative’). They were primarily Caucasian (*n* = 243, or 77.39%) and largely non-religious (*n* = 130, or 41.40%), as opposed to weakly (21.66%), moderately (21.34%) or strongly (15.29%) religious. Of those participants who reported their income (*n* = 121) the large majority (*n* = 96, or 79.34%) reported a yearly income of less than USD 70,000, and a sizeable minority (*n* = 45, or 37.19%) reported a yearly income of less than USD 30,000.

### Study 3

We recruited 100 participants via Prolific Academic. 7 participants were excluded because they failed attention checks or completed the survey too fast. Of the remaining 93 participants, 54 identified as men and 36 identified as women, with 3 participants not providing any response. The mean age was 29.56 years (*SD* = 11.43). Participants were generally politically slightly left leaning (*M* = 3.72, *SD* = 1.43, where 1 = ‘Liberal’ and 7 = ‘Conservative’), economically centrist (*M* = 4.07, *SD* = 1.58, where 1 = ‘Liberal’ and 7 = ‘Conservative’) and socially slightly left-leaning (*M* = 3.61, *SD* = 1.62, where 1 = ‘Liberal’ and 7 = ‘Conservative’). They were primarily Caucasian (*n* = 64, or 68.82%) and largely non-religious (*n* = 44, or 47.31%), as opposed to weakly (25.81%), moderately (19.35%) or strongly (7.53%) religious. The large majority of participants (*n* = 87, or 93,55%) reported a yearly income of less than USD 70,000, and a majority (*n* = 64, or 68.82%) even reported a yearly income of less than USD 30,000.

## Appendix B: additional analyses

### Study 1

**Table:**

Wilcoxon signed-rank test (against the scale midpoint)					
	Name	Mean	Statistic	*p* value	Note
F01	Serious harm	− 0.13	4736	0.478	Not significant
F02	Autonomy	− 0.72	2452	< 0.001	
F03	Rationality	− 0.61	2762	< 0.001	
F04	Social functioning	− 0.64	2838	< 0.001	
F05	Survival	− 1.04	1966	< 0.001	
F06	Flourishing	0.31	5686	0.036	
F07	Irreducibility	0.48	5972	0.002	Not reversed
F08	Normativity	− 1.28	941	< 0.001	
F09	Intra-cultural universality	− 1.54	819	< 0.001	
F10	Cross-cultural universality	− 1.36	1143	< 0.001	
F11	Fallibility	0.68	5204	< 0.001	Not reversed
F12	Objectivity (individual)	− 0.34	3760	0.012	
F13	Objectivity (culture)	− 0.72	2236	< 0.001	

### Study 2

**Table:**

One-sample proportion tests (against the null probability of 0.5)							
		Actual			Modal		
		Proportion	Chi-square (*df* = 1)	*p* value	Proportion	Chi-square (*df* = 1)	*p* value
Goal feature	Serious Harm	**0.31**	22.31	< 0.001	0.43	2.55	0.110
	Autonomy	0.49	0.01	0.936	**0.37**	10.19	0.001
	Rationality	0.52	0.16	0.689	0.42	3.67	0.055
	Social functioning	*0.58*	4.01	0.045	**0.34**	15.92	< 0.001
	Survival	*0.74*	36.06	< 0.001	*0.73*	33.02	< 0.001
	Flourishing	*0.65*	14.16	< 0.001	0.54	0.92	0.338
Non-goal feature	Irreducibility	*0.95*	123.85	< 0.001	*0.81*	58.7	< 0.001
	Normativity	*0.65*	14.16	< 0.001	0.54	0.64	0.425
	Intra-cultural universality	**0.29**	25.44	< 0.001	**0.15**	77.07	< 0.001
	Cross-cultural universality	**0.33**	18.01	< 0.001	**0.12**	88.69	< 0.001
	Fallibility	*0.87*	84.78	< 0.001	*0.9*	97.94	< 0.001
	Objectivity (individual)	**0.18**	62.83	< 0.001	**0.11**	94.8	< 0.001
	Objectivity (culture)	**0.21**	53.08	< 0.001	**0.17**	68.89	< 0.001

Bold values stand for proportions significantly lower than 0.5, while Italic values do for higher than 0.5

## Footnotes

1 Both needs in general and basic needs in particular have of course been extensively studied in psychology before. For example, several researchers have recently empirically tested Maslow’s famous “hierarchy of needs” (e.g., Saeednia & Nor, 2013; Tay & Diener, 2011). Yet, these studies typically do not bear in any sufficiently direct way on attempts to *define* basic needs. They do not specifically investigate potential necessary or characteristic features of this concept; they do not proceed by presenting subjects with cases that may prompt conceptual intuitions; etc.

2 The studies’ registrations are accessible via the following anonymized links: Study 1: https://osf.io/t3yhz/?view_only=f0cad72ba22e4c098b82cebc86f4e194. Study 2: https://osf.io/ec3ar/?view_only=3acedf4710a84434970bc4c65fcafad9. Study 3: https://osf.io/yjwdq/?view_only=3a95107b92484bf39ba179f89fde8df9. All studies were approved by the IRB of the first author’s university.

3 Even some philosophers agree that at least what it means for needs to be “basic” does not have to conform to folk intuitions (e.g., Wiggins, 1998).

4 We would like to thank one of the paper’s anonymous reviewers for pressing us to further clarify the relation between the folk intuitions and the stipulations approach, as we understand them, and be more cautious in our critique of the latter.

5 Our study included two scenarios, aimed at measuring endorsement of F7 = irreducibility and F11 = fallibility, that worked the other way round, i.e., the stronger people agreed with the final statements the more likely they endorsed the features.

6 This focus on basic needs, as opposed to their satisfiers, also clearly emerged in Study 3, when participants were asked to freely associate anything that comes to their mind when they think of the concept (see Table 1).

7 There is also additional independent evidence for our assumption that the concept of basic needs is sufficiently prevalent in ordinary discourse. For example, Google now delivers 45,700,000 results in response to the query “basic needs” (as opposed to, e.g., only 258,000 for “basic capabilities”). And its Ngram Viewer shows that “basic needs” has become a common term in books starting from the late 1930s (with a clear peak around 1980).

8 We would like to thank one of our reviewers for raising this point.

9 We refer to Cohen’s *d* for effect size (Cohen, 1988, 1992). In general, the strength of effect is judged based on a common standard: small effect, *d* = 0.20; moderate effect, *d* = 0.50; large effect, *d* = 0.80. However, note that the criteria should differ across research domain, and there is not a single absolute demarcation regarding effect size.

10 This claim about the concept’s internal structure is also supported by the fact that even though endorsements in Studies 1 and 2 of this paper varied across features, for the most part they were rather low (especially in Study 1), suggesting that even the more strongly endorsed features are only seen as characteristic, not as necessary.

11 Streeten at some points seems to subscribe to the full-life account as well. For example, in (1979: 136) he writes “The objective of a basic needs approach is to provide opportunities for the full development for the individual”. Streeten’s actual lists of basic needs, however, reflect a very narrow conception. In Hicks and Streeten (1979), for example, he only acknowledges health, education, food, water supply, sanitation and housing. In fact, in normative contexts even needs as such (including non-basic needs) have rarely been linked to the idea of a good life. There are only few exceptions that we are aware of (e.g., Anscombe 1958; Grix and McKibbin 2015). In non-normative contexts broad conceptions of basic needs can be found more often. For example, Ryan and Deci (2017) have argued that the basic psychological needs for autonomy, competence and relatedness must be fulfilled for people to be able to flourish in the sense of bringing their possibilities to fruition (see also, e.g., Fiske 2004).

12 Our research provides some initial data about correlations between people’s endorsement of flourishing and their endorsement of normativity. While the correlation was weak in Study 1 (spearman’s *ρ* = .21, *p* = .009), the association was not observed in Study 2 (actual: *χ*2 (1) = 0.21, *p* = .64, *φ* = .037; modal: *χ*2 (1) = 0.66, *p* = .42, *φ* = .065).

13 For this problem see Elster 1983.

14 This also holds for statements about needs-relevant distributive contexts in particular. For example, in the study by Pölzler and Wright (2020), 88% of participants interpreted the statement “A world in which wealth is distributed equally is more just than a world in which it is distributed unequally” as non-objective.

15 We would like to thank an anonymous reviewer for encouraging us to include this point.
